# Cytotoxic and Apoptotic Effects of Different Extracts of *Artemisia turanica* Krasch. on K562 and HL-60 Cell Lines

**DOI:** 10.1155/2013/628073

**Published:** 2013-10-28

**Authors:** Zahra Tayarani-Najaran, Mahla Sareban, Atefeh Gholami, Seyed Ahmad Emami, Mahdi Mojarrab

**Affiliations:** ^1^Department of Pharmacodynamics and Toxicology, School of Pharmacy, Mashhad University of Medical Sciences, Mashhad 9188617871, Iran; ^2^Department of Pharmacognosy, School of Pharmacy, Mashhad University of Medical Sciences, Mashhad 9188617871, Iran; ^3^Novel Drug Delivery Research Center, School of Pharmacy, Kermanshah University of Medical Sciences, Kermanshah 6734667149, Iran

## Abstract

*Artemisia* is an important genus of Iranian flora. Cytotoxic activities for some species of the genus have already been reported. In this study, we have investigated the cytotoxic effects of *n*-hexane, CH_2_Cl_2_, EtOAc, EtOH, and EtOH/H_2_O (1 : 1) extracts of *A. turanica* Krasch. on two human leukemic cancer cell lines (K562 and HL-60) and J774 as normal cells using alamarBlue (resazurin) assay. PI staining of the fragmented DNA and western blot analysis were used to evaluate the possible apoptotic effect of the extract. The CH_2_Cl_2_ extract of *A. turanica* showed the most antiproliferative effect on cancer cells among all tested extracts with IC_50_ values of 69 and 104 **μ**g/mL on K562 and HL-60 cells, respectively, whereas the normal cells were not affected significantly by this extract. Sub-G1 peak in the flow cytometry histogram of the cells treated with CH_2_Cl_2_ extract of *A. turanica* and cleavage of PARP protein confirmed the induction of apoptosis with CH_2_Cl_2_ extract. Taken together, the findings of the present work suggest the anticancer potential of CH_2_Cl_2_ extract of *A. turanica* on human leukemic cancer cell lines.

## 1. Introduction

Cancer is a leading cause of life-threatening disease with limited efficient therapies [1]. Considering the significant levels of toxicity and drug resistance of current anticancer regimens, the challenge to develop highly effective drugs with little or no side effects is crucial. 

Exploring the anticancer ability of novel compounds including plant derivatives provides a wealthy source of novel and potent bioactive compounds with minimal side effects. *Artemisia* is a promising natural source of phytochemicals with potent antimalarial and anticancer properties [2–7]. 

One of the largest genera in the tribe Anthemideae of the Asteraceae (Compositae) is the genus *Artemisia*, which grows mostly in the temperate zone of Asia, Europe, and North America [8]. There are 43 species of the genus in Iran [9], of which two are endemic [10]. Diverse chemical components in this genus such as flavonoids, coumarins, sterols, polyacetylenes, monoterpenes, sesquiterpenes, and sesquiterpene lactones have been reported so far [11, 12]. *Artemisia turanica* Krasch. with the Persian name of “Dermaneye ghermez” grows wildly in northeastern Iran [13]. One study has proved the effect of methanol extract of the aerial parts of the plant against *Staphylococcus aureus*, *Bacillus subtilis *and *Pseudomonas aeruginosa* [14]. Major volatiles from the aerial parts of *A. turanica* were identified as 1,8-cineole, chrysanthenone, and davanone [15]. Antimalarial activity of *A. turanica *during early infection as well as its *in vitro* leishmanicidal activity has been reported [16–18]. The crude hydroethanolic extract showed moderate toxicity against the HepG2 cell line [19].

In an effort to evaluate the potential anticancer effect of different extracts of *A. turanica* on human cancer cell lines, we have investigated the possible cytotoxic activity of the *n*-hexane, CH_2_Cl_2_, EtOAc, EtOH, and EtOH/H_2_O (1 : 1) extracts of *A. turanica* Krasch. on two human leukemic cancer cell lines (K562 and HL-60) and J774 as normal cells. Meanwhile, the possible mechanism(s) of the apoptosis mediated by the plant was also explored. Appearance of the apoptosis related protein and cleavage of PARP provided the first evidence that CH_2_Cl_2_ extract of *A. turanica *could induce apoptosis in human leukemia cells.

## 2. Methods

### 2.1. Reagents and Chemicals

AlamarBlue (resazurin) was obtained from Sigma (Saint Louis, MO, USA); RPMI-1640 and FCS were from Gibco; *β*-actin and PARP antibodies, anti-rabbit IgG, and HRP linked antibody were from Cell Signaling technology (Boston, USA); ECL Western blotting detection reagent was from Bio-Rad (USA); the fluorescent probe propidium iodide (PI), protease inhibitor cocktail, phosphatase inhibitor cocktail, sodium citrate, Triton X-100, phenylmethylsulfonyl fluoride, and Bio-Rad Protein Assay Kit (Hercules, CA, USA) were used; all the solvents used for extraction were purchased from Caledon and Scharlau.

### 2.2. Plant Materials

Aerial parts of the plant were collected from Sami' abad, Torbat Jam (Razavi Khorasan province, Iran) in September 2010. Sample was identified by Dr Valiollah Mozaffarian (Research Institute of Forest and Rangelands, Tehran, Iran). The voucher specimen (no. 12572) has been deposited in the herbarium, Department of Pharmacognosy, School of Pharmacy, Mashhad University of Medical Sciences, Mashhad, Iran.

### 2.3. Preparation of Extracts and Fractions

Air-dried and ground aerial parts (150 g) of* A. turanica *were extracted with *n*-hexane (40–60), CH_2_Cl_2_, EtOAc, EtOH, and EtOH/H_2_O (1 : 1 v/v), respectively (Sequential maceration with ca. 3 × 1.5 L of each solvent). The extracts were filtrated with filter paper and dried using rotary evaporator at a reduced pressure at a temperature below 45°C to yield 4.21, 18.25, 0.91, 5.94, and 28.26 g of each extract, respectively.

**Figure 1:**
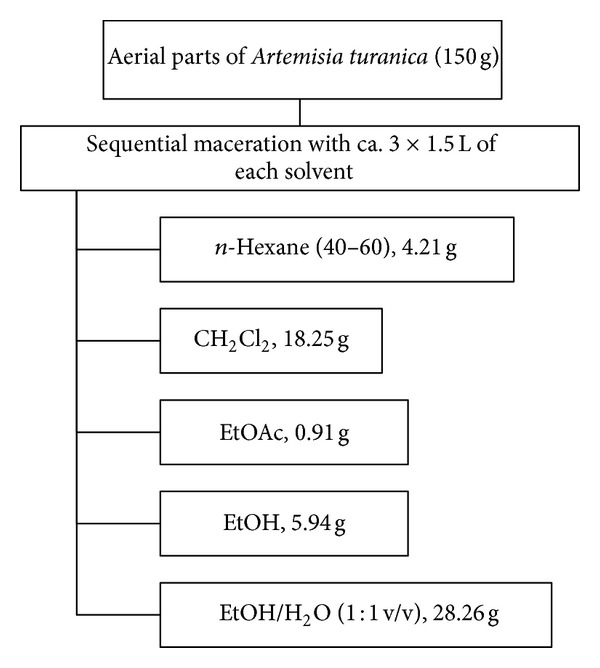
Extraction scheme of *n*-hexane, CH_2_Cl_2_, EtOAc, EtOH, and EtOH/H2O (1 : 1) extracts of *A. turanica*.

All of the isolated extracts were dissolved in dimethylsulfoxide (DMSO) and then were subjected to cytotoxic and apoptosis assays (Figure 1). 

### 2.4. Cell Culture and Treatment Agents

The human leukemic cancer cell lines HL-60 and K562 were obtained from Pasteur Institute (Tehran, Iran) and maintained in RPMI-1640 medium with 10% v/v fetal bovine serum and 100 *μ*/mL penicillin and 100 mg/mL streptomycin at 37°C in a humidified atmosphere of 5% CO_2_ and 95% of air.

### 2.5. *In Vitro* Cell Proliferation

The AlamarBlue reagent is a cell viability indicator using the reducing power of living cells to quantify the proliferation of various cell lines, bacteria, plant, and fungi that allow to measure cytotoxicity of various chemicals. Upon entering cells, the blue and nonflorescent resazurin converts to the florescent and purple resorufin in viable cells [20].

About 5 × 10^4^ K562 and 10^5^ HL-60 cells were seeded in each well of 96-microwell plate and treated with various concentrations of each extract of *A. turanica *(0–200 *μ*g/mL). J774 cell line was used as nonmalignant cells. After 48 incubations, 20 *μ*L resazurin (0.01% w/v in PBS) was added to each well, and the plates were incubated at 37°C for 4 h before the absorbance was measured at 570 nm (test wavelength) and 600 nm (reference wavelength) in a Synergy H4 Hybrid Multi-Mode Microplate Reader (BioTek, Winooski, USA; Winooski is a city in Chittenden). The cytotoxicity of the *A. turanica *extracts was expressed as IC_50_, calculated using Prism 5 Software (GraphPad, La Jolla, CA, USA), and presented as mean ± SEM from three independent experiments (with three replicates for each concentration tested extract). For each study, a control sample remained untreated and received only medium in place of the text materials.

### 2.6. PI Staining

Apoptotic cells were detected by PI staining of small fragments of DNA in treated cells followed by flow cytometry. It has been reported that following DNA fragmentation the so-called sub-G1 peak can be noticed following incubation of cells in a hypotonic phosphate-citrate buffer containing quantitative DNA-binding dye such as PI. Apoptotic cells that have lost DNA will take up less stain and will show up in the left side of the G1 peak in the histogram. Briefly, 10^6^ K562 and HL-60 cells were seeded in each well of a 24-well plate and treated with CH_2_Cl_2_ extract of *A. turanica *in different concentrations (25, 50 and 100 *μ*g/mL) for 48 h. Floating and adherent cells were then harvested and incubated at 4°C overnight in the dark with 750 *μ*L of a hypotonic buffer (50 *μ*g/mL PI in 0.1% sodium citrate plus 0.1% Triton X-100) before flow cytometric analysis using a FACScan flow cytometer (Becton Dickinson, San Diego, CA) was performed. A minimum of 10^4^ events were acquired for each sample. All data were then analyzed using WinMDI Version 2.8 software.

### 2.7. Western Blotting Analysis

About 10^7^ HL-60 and K562 cells were treated with 25, 50, and 100 *μ*g/mL of the CH_2_Cl_2_ extract of *A. turanica *for 48 h. The cells were rinsed and harvested with cool PBS for 3 times; the cell pellet was resuspended in a lysis buffer containing 50 mM tris-HCl (PH 7.4), 150 mM NaCl, 1% TritonX-100, 1 mM EDTA, 0.2% SDS, 1% Protease inhibitor cocktail, 1% phosphatase inhibitor cocktail, and 1 mM phenylmethylsulfonyl fluoride and left on ice for 30 min. After centrifugation at 10000 rpm for 20 min at 4°C, the cell lysate was collected, and protein concentration was determined according to the Bio-Rad Protein Assay kit. Equal amounts of proteins were subjected to 8% and 12.5% SDS-page (W/V). The proteins were transferred to a polyvinylidene fluoride (PVDF) membrane and subjected to immunoblotting using Bax, *β*-actin, and PARP antibody as primary antibodies and anti-rabbit IgG and HRP-linked antibody as secondary antibodies; Bax protein band and PARP cleavage in K562 and HL-60 cells were detected by enhanced chemiluminescence using the ECL western blotting detection reagent. Images were quantified using Gel-pro Analyser V.6.0 Gel Analysis software (Media Cybernetics, Inc, Bethesda, MD, USA).

### 2.8. Statistical Analysis

One way analysis of variance (ANOVA) and Bonferroni post hoc test were used for data analysis. All the results were expressed as mean ± SEM, and *P* values below 0.05 were considered statistically significant.

## 3. Results 

### 3.1. Cytotoxicity of Various Fractions

**Figure 2:**
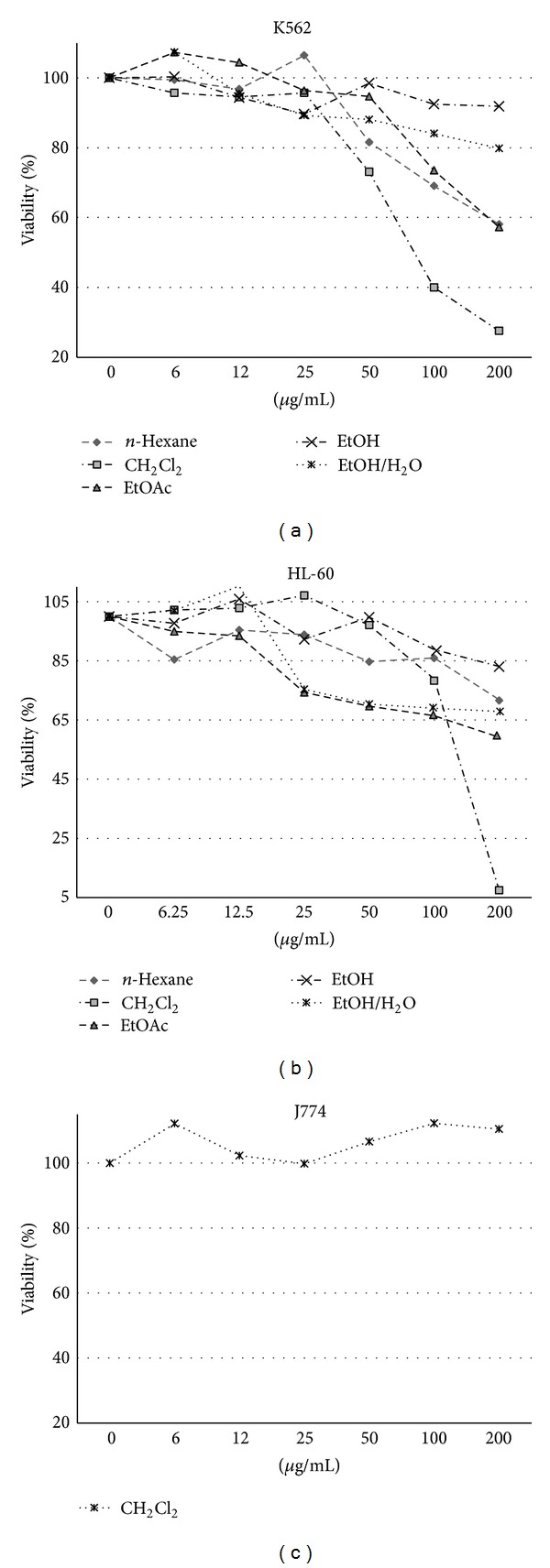
The dose-dependent effects of *n*-hexane, CH_2_Cl_2_, EtOAc, EtOH, and EtOH/H2O (1 : 1) extracts on the growth of K562 and HL-60 cells and normal J774 cells. All extracts exhibited cytotoxic activity against apoptosis-proficient HL-60 and apoptosis-resistant K562 cells, with IC_50_ values ranging from 68.83 to >450 *μ*g/mL and with much less cytotoxic effects on normal J774 cells. Values were mean ± SEM of at least three independent experiments, each in triplicates.

*n*-Hexane, CH_2_Cl_2_, EtOAc, EtOH, and EtOH/H_2_O (1 : 1) extracts of *A. turanica *were examined for cytotoxic potential on K562, HL-60, and normal cells (J774). Cells were incubated at 37°C and 5% CO_2_ with various concentrations of the extract (0–200 *μ*g/mL) for 48 h. Results demonstrated that extracts decreased cell viability in a concentration-dependent manner (Figure 2). Among all the samples, CH_2_Cl_2_ extract demonstrated the most cytotoxic effects on cancer cells but limited adverse effect on normal cells. IC_50_ values (*μ*g/mL) for different extracts of *A. turanica *in HL-60 and K562 cells are presented in Table 1. 

### 3.2. Apoptosis Induction by CH_2_Cl_2_ Fraction

**Figure 3:**
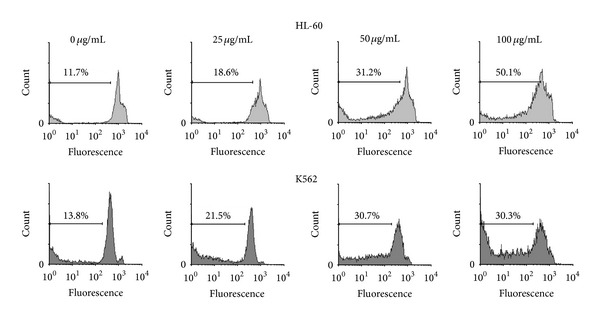
PI staining and flow cytometry analysis of CH_2_Cl_2_ extract (0, 25, 50, and 100 *μ*g/mL) induced apoptosis in K562 and HL-60 cells.

Apoptosis in K562 and HL-60 cell lines was detected with flow cytometry using PI staining test. Cells incubated with various concentrations (0, 25, 50 and 100 *μ*g/mL) of CH_2_Cl_2_ extract of *A. turanica* for 48 h. Sub-G1 peak of treated cells in flow cytometry histograms compared to that (Sub-G1 peak) of untreated control cells revealed the induction of apoptosis in treated cells (Figure 3).

### 3.3. Western Blotting with HL-60 and K562 Cells

**Figure 4:**
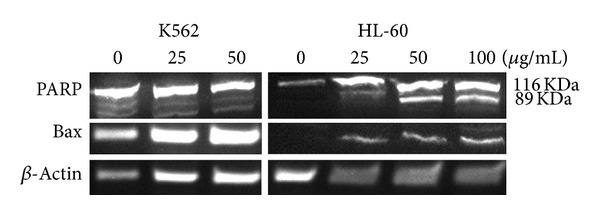
Proteolytic cleavage of poly(ADP-ribose) polymerase (PARP) in HL-60 cells and level of Bax protein in HL-60 and K562 cells after 48 h exposure to CH_2_Cl_2_ extract of *A. turanica* (25, 50 and 100 *μ*g/mL). *β*-Actin was used as a loading control. All Western blots were representative of 3 independent experiments.

The cleavage of 116 kDa PARP-1 to 89 and 24 kDa fragments was used as an indicator of apoptosis. In HL-60 cells, PARP-1 was cleaved clearly to the 89 kDa and 24 kDa fragments after treatment with CH_2_Cl_2_ extract (25, 50 and 100 *μ*g/mL) after 48 h (Figure 4). Bax proteins possess a crucial function in controlling cytochrome c release and apoptosis initiation via the mitochondrial pathway. CH_2_Cl_2_ extract (25, 50 and 100 *μ*g/mL) could not change the level of Bax protein in both cells (Figure 4).

## 4. Discussion

Strong evidence supports the critical role of apoptosis in the pathology of many diseases including cancer. Thus, pharmacological modulation of apoptosis is likely to be the main strategy for searching for efficient anticancer therapeutics [21].

The result of the present study supports the cytotoxic and apoptotic activity of CH_2_Cl_2_ extract of *A. turanica* when compared with other extracts obtained from the plant on two human leukemic cancer cell lines (K562 and HL-60). 

Using *n*-hexane, CH_2_Cl_2_, EtOAc, EtOH, and EtOH/H_2_O (1 : 1) solvents for extraction would afford different fractions extracts that contain the different groups of phytochemicals corresponding to the various polarity of the extractant [22]. Comparison of the results obtained with different extracts of the *A. turanica *confirmed the presence of potent non/semipolar phytochemicals in CH_2_Cl_2_ extract of the plant. 

Apoptosis induction was validated using PI staining of fragmented DNA and western blot analysis of the proteins involved in programmed cell death pathway. PARP cleavage as an important indicator for apoptosis induction was consistent with other results obtained in this study. The unchanged level of Bax protein in K562 cells may reject the role of mitochondria in the apoptosis pathway. Full analysis of the proteins involved in the intrinsic pathway helps to recognize the role of the extract in apoptosis induction in cells.

Death receptor and mitochondria initiated apoptosis recruit caspases as the crucial enzyme in cell death. Caspase 8 and caspase 9 activation merged to caspase 3 stimulation, which leads to changes in the activity of some of the important enzymes involved in DNA repair. Cleavage of PARP is one of the examples of enzyme inactivation in apoptosis, which leads to unrepaired single-strand DNA breaks that accumulate in the absence of PARP activity [23].

The overcome of proapoptotic proteins like Bax to antiapoptotic proteins, located on the outer layer of the mitochondria, opens pores on the surface of the mitochondria leading to the release of cytochrome c, apoptosome formation, and caspase activation [24].

Due to some intrinsic differences, the cytotoxic results on HL-60 and K562 cells used in this study were different. The absence of the Fas/CD95/APO-1 receptor in K562 cells may be the main reason for lower IC_50_s in this cell line [25]. Accordingly, apoptosis was induced in lesser extent in apoptosis-resistant K562 cells when compared with apoptosis-proficient HL-60 cells. Since the role of mitochondria in the apoptosis-induction of the plant has not been proven, interaction of the extract with death receptors other than the Fas/CD95/APO-1 receptor in K562 cells has been speculated. 

Plants serve as the important part of the antitumor regimen both in conventional and alternative medicine. Many plants of the genus *Artemisia* have been reported to possess promising effects in research also in treating cancer [26, 27].

A cytotoxic evaluation of the isolated dimeric guaianolides from *A. anomala* showed significant inhibitory effects against the cell growth of BGC-823 tumor cell lines [28]. Two new eudesmane sesquiterpenoids from the same species exhibited cytotoxicity against HCT-8 and A549 cell lines [29]. 5/7-fused bicyclic guaianolides isolated from *A. myriantha* and *A. absinthium* are classified in one of the major categories of *α*-methylene-*γ*-lactones with anticancer potential [30]. A naturally occurring flavonoid, eupatilin, isolated from *A. princeps* inhibited the growth of human endometrial cancer cells via G2/M phase cell cycle arrest [31]. RXF-393 renal cancer cell line displayed high sensibility to the organic extract from the leaves of *A. verlotiorum,* which induced a significant dose-dependent increase in the lipid peroxidation [32]. Drimartol A, a sesquiterpene coumarin ether, and two other new sesquiterpenes could efficiently induce apoptosis of a human lung cancer cell line (95-D) through the mitochondrial-dependent pathway. The compounds were isolated from the cultured hairy roots of *A. annua* [33–35]. Isoscopoletin from *A. argyi* and artemisinin from *A. annua* have shown great cytotoxicity against lung and colon cancers [35]. A sesquiterpene lactone purified from *A. diffusa* inhibited spontaneous mouse mammary tumor growth *in vivo* [36]. The essential oil of *A. capillaris* is believed to be a good resource for searching new drugs, especially anticancer drugs because of its ability to induce apoptosis in human oral cancer cells [37].

The biological evaluation of the whole plant is provided to assess the synergistic and antagonistic interactions of mixture of phytochemicals existing in the extract [38].

Taken together, cytotoxicity and DNA fragmentation along with cleavage of PARP confirmed the apoptotic activity of the CH_2_Cl_2_ extract of *A. turanica*. These results indicated the presence of non/semipolar nature of the phytochemical responsible for the observed effects. Further analytical experiments on CH_2_Cl_2_ extract of *A. turanica* and structure elucidation should be performed to recognize the pure component responsible for the cytotoxic activity of the plant.

## Figures and Tables

**Table 1:** IC_50_ values (*μ*g/mL) for different extracts of* A. turanica *in HL-60 and K562 cell lines.

Cell line	Fractions				
	CH_2_CI_2_	*n*-Hexane	EtOAc	EtOH	EtOH/H_2_O (1 : 1)
K562	104.2	234.5	433.1	>450	>450
HL-60	68.83	>450	373.7	>450	>450
